# Influence of the Physical State of Two Monofloral Honeys on Sensory Properties and Consumer Satisfaction

**DOI:** 10.3390/foods12050986

**Published:** 2023-02-26

**Authors:** Maria Lucia Piana, Marta Cianciabella, Giulia Maria Daniele, Anna Badiani, Pietro Rocculi, Silvia Tappi, Edoardo Gatti, Gian Luigi Marcazzan, Massimiliano Magli, Chiara Medoro, Stefano Predieri

**Affiliations:** 1 Piana Ricerca e Consulenza, Castel San Pietro Terme (BO), 40024 Bologna, Italy; 2 IBE-Institute of BioEconomy, CNR, Via Gobetti, 101, 40129 Bologna, Italy; 3 Veterinary Medical Sciences Department, University of Bologna, 40126 Bologna, Italy; 4 Agricultural and Food Sciences Department, University of Bologna, 40126 Bologna, Italy; 5 CREA Council for Agricultural Research and Economics, Agriculture and Environment Research Center, 40128 Bologna, Italy

**Keywords:** honey quality, honey sensory analysis, crystallization, consumer’s preferences, CATA, monofloral honeys

## Abstract

Honey is a worldwide known and appreciated food product. Its appreciation by consumers is due to both its nutritional properties and the extremely reduced processing. The floral origin, color, aroma and taste are key factors in determining the quality of honey. Nevertheless, rheological properties, as crystallization rate, play a fundamental role in the perceived overall quality. Indeed, crystallized honey is often considered of poor quality by consumers, but a fine-grained or creamy texture is becoming interesting from the producers’ side. The purpose of this study was to investigate textural and aromatic properties and consumers’ perception and acceptance of two monofloral honeys that were differently crystallized. Liquid and creamy samples were obtained from crystallized samples. Physico-chemical, descriptive and dynamic sensory analysis, as well as consumer and CATA tests, were conducted on the three honey textures. The physico-chemical analysis well-discriminated the crystallization levels and evidenced that, although the honey variety was different, the textural properties of the creamy samples are very similar. Crystallization was shown to affect the honey sensory perceptions: liquid samples were sweeter, but less aromatic. Consumer tests allowed the validation of panel data and confirmed consumers’ higher appreciation for liquid and creamy honey.

## 1. Introduction

Honey is the most important product of apiaries and is worldwide consumed, making it economically important [1]. Its consumption has been rising thanks to its nutritional benefits and due to health consciousness and concerns focused on food processing technologies [2,3] since its production is not subjected to any technological processes [4,5,6].

As with any food product, honey must meet safety and quality criteria. Honey’s quality criteria are defined by the Codex Alimentarius standard [7] and the EU Honey Directive [8]. Those criteria describe the honey on the basis of its composition through the quantification of the water and sugar content, reducing sugars, proteins, minerals, calories, ashes, free acidity, insoluble solids, enzymes, electrical conductivity, color, as well as antibiotics and pesticides residues [1]. Many of those characteristics, as well as physical properties, such as pH, acidity, viscosity, electrical conductivity, and color, also depend on the honey type (monofloral/polyfloral) and origin [3]. Nevertheless, instrumental analyses are not sufficient to define the perceived quality, thus a comprehensive quality evaluation of the sensory and analytical criteria is considered to be the best method to evaluate honey quality [9,10,11].

Since composition, color, aroma and taste are key factors in determining honey quality [10], the rheological properties constitute one fundamental physical feature determining its quality [12,13]. Honey is a highly viscous sugar solution, often supersaturated and susceptible to time-dependent crystallization, at a rate influenced by the water content, presence of nucleation seeds, degree of supersaturation and viscosity that are in turn related to temperature. While some monofloral honeys (e.g., citrus) are naturally crystallized, for most of the commercial honeys, crystallization is a defect. Indeed, spontaneous granulation can lead to undesired coarse crystals and may cause quality loss due to phase separation, sedimentation and water activity, which can promote microbial fermentative processes [14].

Honey crystallization, also defined as granulation, influences consumers’ preferences [15,16], making it less appealing to consumers, who prefer liquid and/or transparent honey [17,18,19]. Crystallization rather affects the perceived quality more than the objective quality, as observed by [20], since many consumers still think that granulated honey has gone bad or has had sugar added [21]. At the same time, there is an increasing interest in fine-grained or creamy honey characterized by a semi-liquid consistency [22].

The aim of this work was to investigate sensory properties and consumers’ perception and acceptance of two different monofloral honeys related to texture properties influenced by processing methods rather than the product origin.

## 2. Materials and Methods

### 2.1. Product Information: Honeys

The study was carried out on two monofloral honeys, citrus (*Citrus* spp.) and rape (Brassica napus) honey, representing different aromatic properties and their related consumers’ acceptability levels. For each monofloral honey, three different physical states (liquid, creamy and crystallized) were considered.

For each monofloral honey, 30 kg of the same crystallized batch, certified according to the chemical–physical and melissopalynological analysis UNI 11299:2008 [23], were recruited from a commercial supplier.

For each monofloral honey, the liquid and creamy samples were obtained from crystallized honey. The liquid sample was obtained by heating 20 kg of the crystallized sample for 24 h at 45 °C [24]. Liquid samples, obtained after melting, were analyzed to confirm that crystals were no longer present in the honeys before beginning the guided crystallization process. The creamy samples was derived by 10 kg of the liquid sample induced granulation using a crystal seed and constant agitation for 4 days at 14 °C.

Table 1 reports the saccharides content: fructose, glucose, sucrose, turanose, and maltose, through the HPLC method [25], and the water content of each honey.

### 2.2. Rheological and Physical Analysis

#### 2.2.1. Differential Scanning Calorimetry (DSC)

DSC mod, Q20 (TA Instrument, Eschborn, Germany) equipped with a cooling unit (TA-Refrigetated Cooling System 90), was used to carry out thermal analysis. Heat flow and temperature calibration were performed with distilled water (Tm 0.0 °C) and indium (Tm 156.60 °C) under a dry nitrogen flow of 50 mL min^−1^. For each sample, honey was weighed in triplicate in 50 μL aluminum DSC capsules and sealed. Samples were scanned at 5 °C/min from 14 to 100 °C.

Peaks were integrated with the Software TA-Universal analyzer determining melting temperature (Tm, °C) and melting enthalpy (ΔH, J/g).

#### 2.2.2. Microscopic Observation

The honey microstructure was evaluated by microscopic observation using an upright Nikon microscope mod: eclipse Ti-U (Nikon Co., Ltd., Tokio, Japan) equipped with a Nikon digital video camera, and digital sight mod; and DS-Qi1Mc (Nikon Co., Ltd., Tokio, Japan) equipped with a polarizing filter at 4× magnification.

#### 2.2.3. Water Activity

Water activity (*a_w_*) was measured at 25 °C using ACQUA LAB Water Activity Meter, mod. CX3-Te (Decagon Devices Inc., Pullman, WA, USA). For each sample, 3 replicates were analyzed.

#### 2.2.4. Texture Measurement

Textural parameters were evaluated through a compression test performed with a Texture Analyzer TA.HDi500 (Stable Micro Systems, Surrey, UK) according to Conforti et al. [26], Tappi et al. [27], and Dettori et al. [28], with some modifications. A cylindrical probe with a flat cross-section (d = 10 mm) at a displacement speed of 0.5 mm/s was used. With the acquired curves of force (N) versus time (s), various parameters were calculated: firmness (N) (the force registered at the highest peak), and adhesivity (N s) (the negative force area obtained after compression). Samples were measured at room temperature (25 °C) in honey pots (80 g). For each sample, 3 replicates were analyzed.

#### 2.2.5. Color Measurement

Color was determined through a spectrophotocolorimeter mod, HUNTER LAB Color-FlexTM (A60-1010-615, Reston, VA, USA) equipped with a sample holder port size 50″. The instrument was calibrated with a black and white standard tile before each set of measurements. For each sample, 3 replicates were analyzed.

### 2.3. Sensory Evaluation

#### 2.3.1. Sample Preparation and Presentation

Sensory analysis and consumer test, were performed by the expert panel and consumers, in individual booths equipped with notebooks running a specific software for sensory data acquisition (FIZZ, Biosystemès, Couternon, France), according to the standard protocol UNI 8589:1990 [29], at the CNR campus sensory lab in Bologna. Samples were presented in a closed glass honey-pot (80 g) labeled with three-digit random numbers, and served on a white plastic tray at room temperature (20+/−2 C°).

#### 2.3.2. Expert Panel

Twelve expert assessors were selected among the Italian Register of Experts in the Honey Sensory Analysis. Each panelist had more than 70 h of training in honey sensory profile analysis. The panelists were also previously trained on dynamic tests (30 min session before each test) to ensure familiarity with time intensity (TI) and temporal dominance of sensations (TDS). Tests performed by the expert panel were carried out in duplicate and presented monadically in a balanced order. Panelists used water and apples to rinse their mouths between samples. Before testing, panelists were informed of the main research outcomes and gave consent for their data to be used. Participation in the research was voluntary, and the right to privacy and data protection was respected in accordance with current legislation (GDPR 2016/679).

#### 2.3.3. Descriptive Analysis (DA)

Fourteen sensory attributes were selected from the literature and used for descriptive analyses. Twelve olfactory and gustatory attributes were chosen: global olfactory intensity (strength of the stimuli perceived by olfactory receptors via nasal and retro-nasal pathway), floral flavor, fruity flavor, warm flavor, aromatic flavor, chemical flavor, vegetal flavor, animal flavor, sweetness, acidity, bitterness and saltiness [30]. Two texture attributes were also included: firmness and grainy (crystal dimension). The descriptors were evaluated through a non-structured 10 cm scale from “no perception” to the “highest intensity perceivable”.

#### 2.3.4. Time Intensity (TI)

TI tests evaluated the global flavor intensity (the stimulus perceived by the olfactory receptor through the retro-nasal path), using an unstructured horizontal scale, from 0, ”no perception”, to 100, “high intensity”. Panelists were requested to take a teaspoon of honey in their mouth, click on “start” and describe the descriptor intensity evolution in their mouth, moving the cursor on the scale until the perception ended, during the whole tasting experience (90 s) [31].

#### 2.3.5. TDS Test

Assessors were instructed to check, among a list of aromatic attributes (floral flavor, fruity flavor, warm flavor, aromatic flavor, chemical flavor, vegetal flavor and animal flavor), the one catching their attention, indicated as dominant [31], for 90 s, the whole duration of the tasting, aftertaste included. The test starts when the assessor puts the sample in their mouth (7 g equivalent to 1/3 tablespoon) and immediately clicks start. When the dominant perception changed, the panelist indicated the new dominant sensation, until the perception ended. Participants were free to choose the same attribute several times or never select an attribute. Attributes’ order was randomized [31] among participants to reduce potential bias due to the attribute position [32]. Data collection was performed through the Fizz software Byosistemes.

#### 2.3.6. Consumer Test

Consumers participated voluntarily according to their interests and availability, they were informed on the main research outcomes and gave consent for their data to be used. The right to privacy and data protection was respected in accordance with current legislation (GDPR 2016/679).

The survey was structured in a different section. First, demographic information and honey consumption habits were requested. Consumers were asked to quantify the honey consumption through the agreement on 8 statements using a 9-point scale, from 1, “disagree completely”, to 9, “agree completely”. The statements included: (i) how I consume honey (as is, spread on a slice of bread, as a sweetener, as an ingredient in food, in food pairing), (ii) why I consume honey (because I like it, for therapeutic use, because it is a “natural/genuine” product). Then, respondents had to test samples, scoring the visual, texture and taste liking. Samples were presented monadically, in a closed glass honey-pot (80 g) labelled with three-digit random numbers, at room temperature. The sample presentation order followed a complete block design balanced for carry-over and position effects. Consumers rinsed their mouths with water between samples.

Liking was scored on a 9-point hedonic scale, from 1, “extremely dislike”; 5, “neither like nor dislike”; to 9, “extremely like”.

Again, the texture was evaluated on a 9-point hedonic scale by stirring honey with a teaspoon. Respondents were also asked to visually describe samples through a CATA test. They had to check, from among a list of 18 visual terms, the ones describing the samples the best. The 18 terms, previously selected through a focus group of experts, were creamy, pearly, homogeneous, brilliant, viscous, spreadable, tender, natural/genuine, thick, fluid, transparent, artificial, limpid, opaque, liquid, hard, grainy, and non-homogeneous.

Finally, consumers had to describe taste through a CATA test and score their overall liking on a 9-point hedonic scale. The 26 CATA terms, previously selected through a focus group of experts, were adhesive, creamy, melting, rough, hard, tender, natural/genuine, artificial, liquid, fine crystals, big crystals, floury, refreshing, balsamic, sweet, persistent flavor, pungent, pleasant flavor, floral, off-flavor, fruity, greasy, chemical flavor, and boiled vegetable. For the term sweet, three intensity adjectives were used (low, optimal, and high) [33,34,35]. The terms’ presentation order was randomized between and within participants. Consumers rinsed their mouths with water between samples.

### 2.4. Statistical Analysis

Data were analyzed using R (R Core Team 2021. R: A language and environment for statistical computing. R Foundation for Statistical Computing, Vienna, Austria. Version 4.1.2) with packages “SensoMineR” version 1.20, “FactoMineR” version 1.36 and “tempR” version 0.9.9.20.

#### 2.4.1. Physical Characteristics

Physical characteristics and sensory profiles of honey samples were analyzed using a one-way ANOVA model and Tukey’s honestly significant differences for post hoc mean separation.

#### 2.4.2. Descriptive Analysis (DA)

Sensory profiles evaluated by the expert panel were submitted to the ANOVA procedure by a complete factorial design (botanical origin, crystallization state and relative interaction) to determine the effect of each factor [36]. Sensory profiles of each unifloral honey sample were analyzed using a two-way ANOVA mixed model with assessors as a random factor and Tukey’s honestly significant differences for post hoc mean separation.

#### 2.4.3. TI Curves

For each sample, average TI curves were generated by averaging the data at each time point across all panelists. No specific averaging method was used [31]. The following parameters were extracted for each individual time intensity sequence: the maximum intensity (Imax; the highest intensity on TI record), the time at the maximum intensity (Tmax; time to reach peak intensity), area under the curve (AUC; total area under the time-intensity curve), and total duration (Dur; the time for the perception of global olfactory intensity from the first to the last perception) [31,37,38,39]. These parameters were analyzed using a one-way ANOVA model, and post hoc comparisons (Tukey’s HSD test) were used to test for differences between the honey samples.

#### 2.4.4. TDS Curves

TDS curves computation considers each attribute separately. For each time point, the proportion of runs for which the given attribute was assessed as dominant was computed. These proportions, smoothed using R with the package “tempR”, were plotted against time and called TDS curves. For each product, the TDS curves of all the attributes were plotted on the same graph [31].

#### 2.4.5. Consumer Data

Visual liking, texture liking and overall liking were submitted to the ANOVA procedure with a complete factorial design (botanical origin, crystallization state and relative interaction) to define the effect of each factor.

A one-way ANOVA model and Tukey’s post hoc mean separation at the 5% significance level were used to determine significant differences in liking and consumption behavior levels.

The citation frequency of each sensory attribute was determined by counting the number of consumers who used that term to describe each honey sample. Cochran’s Q test was carried out to identify significant differences between samples for each of the terms included in the CATA questions. The sign test was used for pairwise comparisons.

Correspondence analysis (CA) was used to obtain a bi-dimensional representation of the samples and the relationship between samples and terms from the CATA question. This analysis was performed on the frequency table containing the samples in rows and the terms from the CATA question in columns.

## 3. Results

### 3.1. Rheological Analysis

#### 3.1.1. Calorimetric Analysis

**Figure 1:**
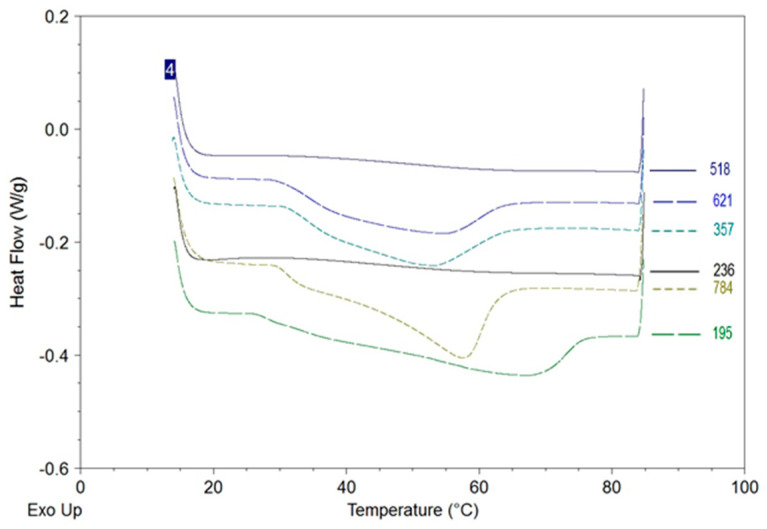
Thermograms of samples obtained by DSC. Citrus honey samples are identified by code 518 (liquid), 621 (creamy), 357 (crystallized). Rape honey samples are identified by code 236 (liquid), 784 (creamy) and 195 (crystallized).

Differential scanning calorimetry has been confirmed to be a suitable technique to evaluate the crystallization state of honey [14,40,41]. Figure 1 shows an example of the samples’ thermograms obtained by DSC analysis.

The presence of endothermic peaks between 27–65 °C represents the melting of glucose crystals, as observed by Lupano [40].

Liquid samples (518 and 236) showed flat base lines with no endothermic peaks, indicating the absence of detectable glucose crystals.

The enthalpy of melting was evaluated for the other samples and reported in Table 2.

Citrus honey samples had values between 21.8 and 22.7 J/g, and rape honey varied between 31.4 and 31.5 J/g. No significant differences were observed between creamy and crystallized samples for both types of honeys, showing that the degree of crystallization, defined as the total amount of crystallized glucose, did not differ. On the contrary, citrus and rape honey had different crystallized glucose content directly related to the sugar content (33.1 and 37.6, respectively).

Citrus honey showed a broad flat peak in the thermograms both for creamy and crystallized samples, with a maximum in the range of 52–53 °C.

Rape honey thermograms showed different shapes, despite having similar enthalpy values, indicating variability in the granulation process. A main peak, about 57 °C, and a shoulder one, about 35 °C, characterized the creamy sample (784). The crystallized sample (195) had its maximum at around 67 °C, with different shoulders appearing at lower temperatures.

#### 3.1.2. Microscopic Analysis

**Figure 2:**
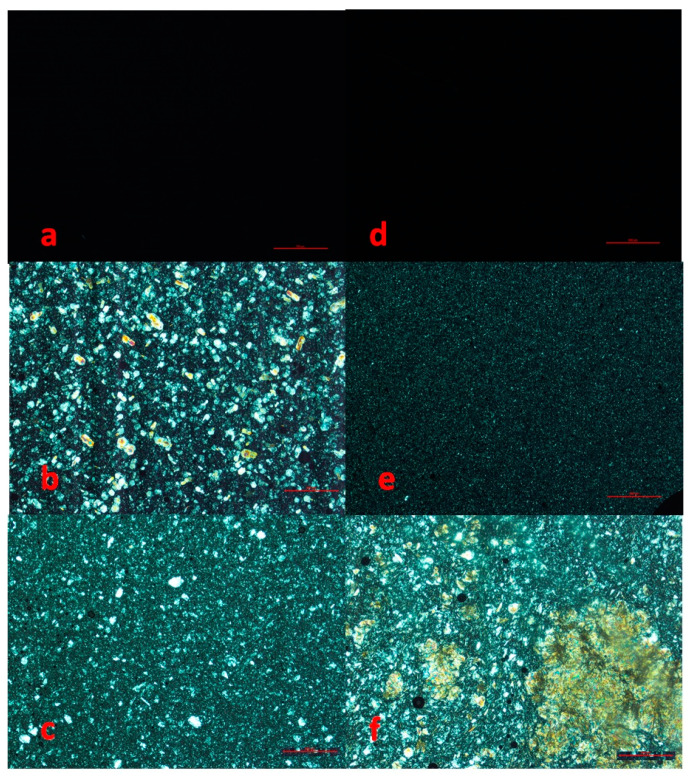
Microscopic images of citrus honey: liquid (**a**), creamy (**b**), and crystallized (**c**). Microscopic images of rape honey: liquid (**d**), creamy (**e**), and crystallized (**f**) at a magnification of 4×. Bars correspond to 500 µm.

Figure 2 shows microscopic images of honey samples taken with polarized light.

Crystals are clearly visible on the black background. Liquid samples (Figure 2a,d) showed the absence of crystals. Creamy samples appeared very different depending on the type of honey considered. Rape honey (Figure 2e) had only very small crystals (maximum diameter < 10 µm), and the citrus sample (Figure 2b) showed high dimension variability. Indeed, 20–50 µm crystals were more represented, and a few bigger crystals (50–100 µm) were visible. Finally, the citrus crystallized sample (Figure 2c) was characterized by medium size crystals (up to 90 µm diameter), and looked similar to the creamy one. On the contrary, rape honey images confirmed a non-homogeneous crystallization, with crystals forming bigger structures along with isolated crystals.

#### 3.1.3. Physico-Chemical Parameters

Liquid honeys were characterized by aw values of 0.515 and 0.530, for citrus and rape honey, respectively (Table 3), which in turn were related to the water content (17.1 and 18.7%, respectively).

The citrus sample showed a 0.03 *a_w_* increase; while the rape honey aw increase was 0.05. Both values were below 0.6, avoiding fermentation; moreover, there were no differences between creamy and crystallized samples.

The analyzed texture parameters, related to the crystallization process, revealed that liquid honey had low adhesivity values (Table 3).

Citrus and rape crystallized samples showed average values of 10.3 and 5.3 N, respectively. The rape sample (195) showed high variability due to a non-homogeneous granulation confirmed by the high standard deviation. On the other hand, creamy samples showed hardness values similar to the liquid ones, indicating the induced granulation, obtained using a crystal seed and constant stirring, allowed them to maintain low hardness, even at full crystallization. Similarly, adhesivity increased in the crystallized sample, but not in the creamy ones for both honey types.

The honey color was evaluated through brightness (L*), red index (a*) and hue angle (h°) (Table 3). Liquid honey showed significant differences between the two honey types, as rape honey was darker and redder than the citrus one. In the creamy sample, there was an increase in a* and a decrease in h°, and the a* increase was even higher in crystallized samples for both honey types. The L* changed according to both the honey type and crystallization. The L* value was stable in both types of honey in crystallized samples, while it decreased in citrus and increased in rape creamy samples.

### 3.2. Sensory Results by the Expert Panel

#### 3.2.1. DA

The DA intensity results are summarized in Table 4.

Significant differences were registered for global olfactory intensity, floral, chemical, vegetal, animal, and sweetness, mainly related to the monofloral origin of the honey. Floral and sweetness attributes showed a moderate but significant effect on the crystallization factor. Firmness was completely associated to the crystallization effect (99.00% of the total variance). Grainy was equally distributed between the two factors and their interaction (Table 5).

Citrus honeys were characterized by a high floral flavor while rape honeys showed typical vegetal, chemical and animal flavors. In citrus honey, the aromatic floral attribute showed significant differences among the crystallization types: 2.18 for the liquid sample, and 3.33 and 3.22 for creamy and crystallized samples, respectively. Sweetness followed the same trend: higher in liquid samples (7.00), and lower in crystallized ones (5.27). A slight increase of sweetness in the fluid samples was noticed in rape samples as well.

Firmness was higher in crystallized samples for both honey types; there were no significant differences between liquid and creamy samples (*p* < 0.0001). Grainy was only perceivable in crystallized and creamy samples. Significant differences were observed (*p* < 0.0001) between different crystallization levels in each monofloral honey type. The crystallized rape sample had the highest grainy intensity scores (8.13), whereas the crystallized citrus sample showed a lower intensity score (0.42), and no significant differences were recorded between crystallized and creamy samples.

#### 3.2.2. TI

**Figure 3:**
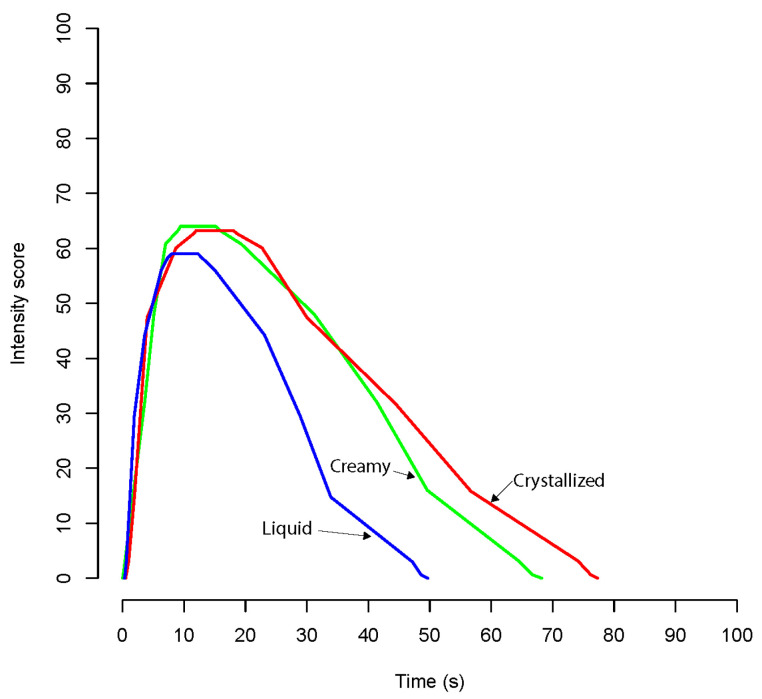
Time intensity curves for citrus honey samples.

**Figure 4:**
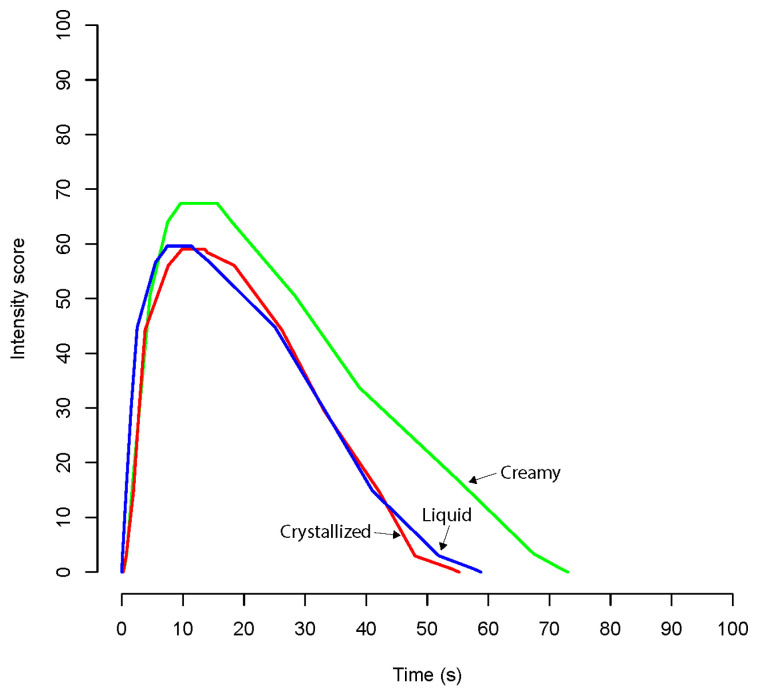
Time intensity curves for rape honey samples.

The TI curves for the overall flavor intensity are shown in Figure 3 and Figure 4.

In citrus honey, the TI curves showed a similar trend for both creamy and crystallized samples: the flavor gradually increased during the tasting phase until reaching a plateau at 10–20 s, then decreased. In liquid samples, the flavor reached the plateau at 10 s and rapidly decreased. In these honey samples, the maximum overall flavor intensity (Imax) did not vary significantly among the crystallization levels, while the time to maximum overall olfactory intensity (Tmax) varied significantly (*p* = 0.0178) between products. Notably, Tmax was significantly longer for the crystallized product than the liquid and creamy ones. Even if the total duration (Dur) did not vary significantly (*p* = 0.0653) between products, the TI curves were shorter for the liquid sample and longer for the crystallized citrus honey sample. The total area under the time-intensity curve (AUC) varied significantly (*p* = 0.0336) between different levels of crystallization. In particular, AUC was significantly lower for the liquid products than the crystallized and creamy (Table 6) ones.

In rape honey, different crystallization levels showed similar curves (Figure 4). However, the Tmax was significantly shorter for the liquid sample (*p* = 0.0451). The creamy sample showed significantly higher Imax and AUC (*p* = 0.0237 and *p* = 0.0061, respectively). The Dur was also significantly longer than the liquid and crystallized rape honey samples (*p* = 0.0387). The differences in TI curves are summarized in Table 7.

#### 3.2.3. TDS

**Figure 5:**
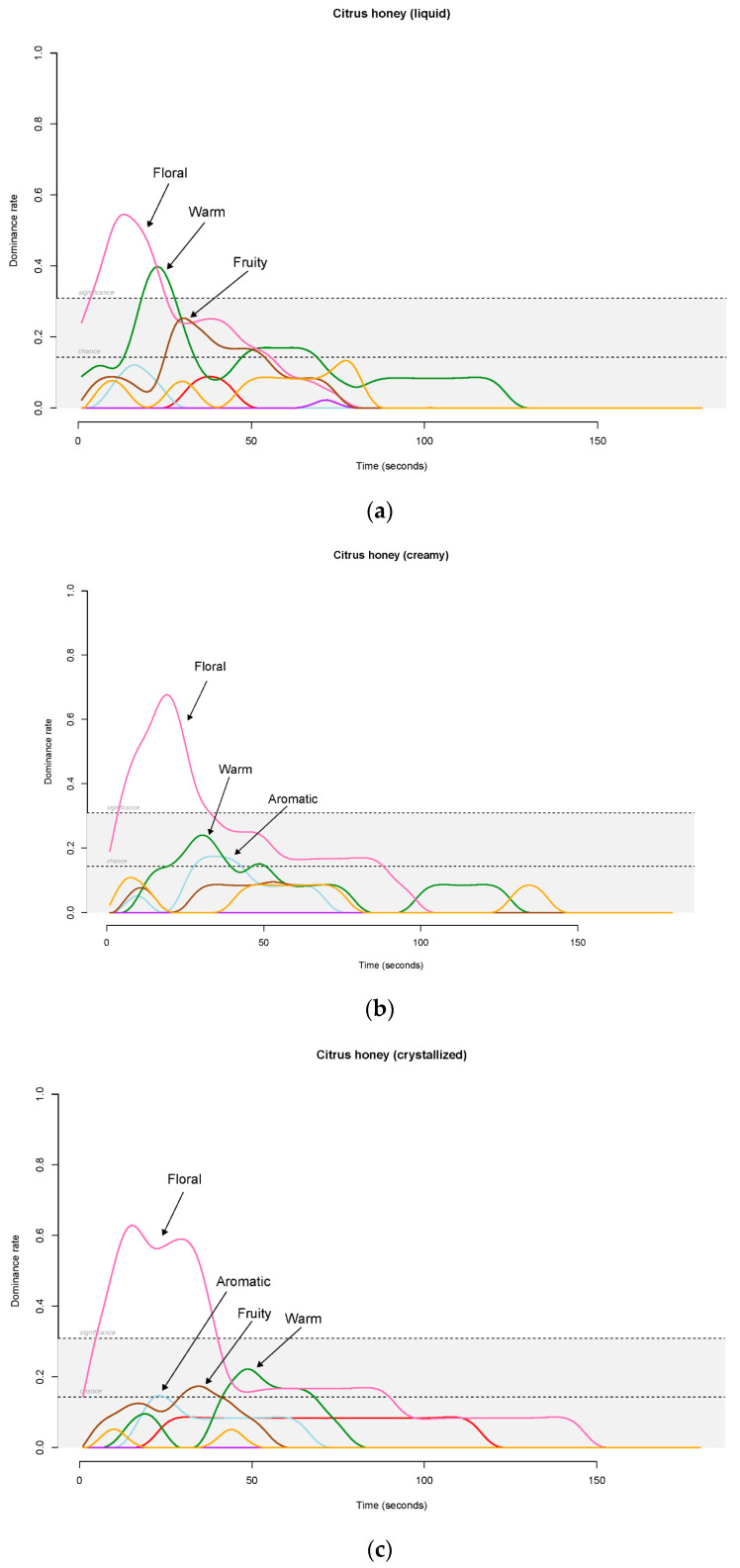
(**a**) TDS graphical representation for the liquid citrus honey. For each attribute, the spline regression plot displays the nonlinear regression function plotted through the original data. (**b**) TDS graphical representation for the creamy citrus honey. For each attribute, the spline regression plot displays the nonlinear regression function plotted through the original data. (**c**) TDS graphical representation for the crystallized citrus honey. For each attribute, the spline regression plot displays the nonlinear regression function plotted through the original data.

The TDS graphs for the three citrus honey samples are represented in Figure 5a–c. The floral flavor was dominant in all the samples. However, liquid citrus honey (Figure 5a) showed a different trend: floral flavor was only dominant for 20 s, and subsequently, the warm attribute appeared.

The dominance duration, for the three kinds of honey, was in line with the TI for the overall olfactory intensity, showing a longer dominance for the crystallized sample (40 s of evaluation).

**Figure 6:**
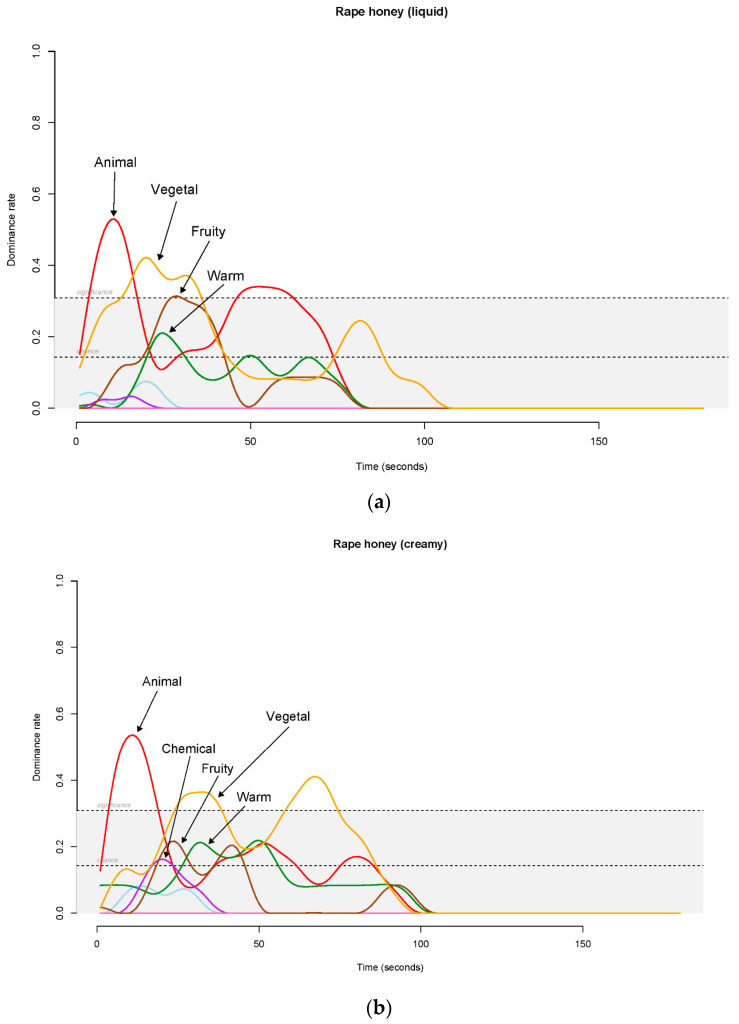

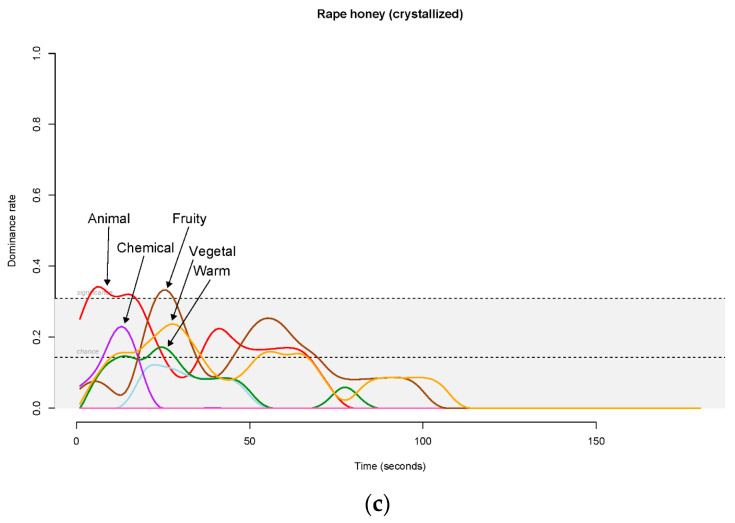
(**a**) TDS graphical representation for the liquid rape honey. For each attribute, the spline regression plot displays the nonlinear regression function plotted through the original data. (**b**) TDS graphical representation for the creamy rape honey. For each attribute, the spline regression plot displays the nonlinear regression function plotted through the original data. (**c**) TDS graphical representation for the crystallized rape honey. For each attribute, the spline regression plot displays the nonlinear regression function plotted through the original data.

The TDS curves for rape honey showed differences among the crystallization levels (Figure 6a–c).

In the creamy sample, the dominant attributes were animal (first 20 s) and vegetal flavors (from 75 s) (Figure 6b). Chemical, fruity and warm flavors appeared during the central part of the tasting phase with dominance rate values greater than the chance level. The crystallized sample was characterized first by animal (first 20 s) and then by fruity (from 20 to 30 s). Chemical, vegetal and warm flavors showed dominance rate values greater than the chance level during the central part of the evaluation time (Figure 6c). The liquid sample (Figure 6a) showed a different trend: animal, vegetal and fruity flavors were dominant for the first half of the tasting, and then warm flavor appeared with a dominance rate value greater than the chance level at 25 s of the tasting. The dominance duration was in line with the TI of the overall olfactory intensity. The creamy sample showed a longer dominance than the liquid and crystallized ones.

### 3.3. Consumer Acceptance

Overall, 139 consumers (66 female, 73 male), aged between 22–67 years old (mean age 40.7; std 12.4) evaluated the six honeys.

Of them, 52% said that they consumed honey at least once per week; 34% said they consumed honey at least once every month, while only 14% consumed honey once every year. Honey was mainly consumed as a sweetener, followed by “in food pairing”, “as is” and “spreaded on bread”. Honey use as an ingredient recorded the lowest agreement level (Table 8).

Reasons for consumption were mainly “I like it” followed by “Natural/Genuine product” and “therapeutic use” (Table 9).

#### 3.3.1. Liking Score

Table 10 shows the impact of the botanical origin, crystallization state and their interaction on visual, texture, and overall liking analyzed by ANOVA factorial design.

Visual and texture liking showed highly statistically significant values for the crystallization factor with 90.54% and 85.54% of variance explicated by the model, respectively. Only less than 10% of variability accounted for the botanical origin and its interaction with crystallization both for visual and for texture liking. The overall liking variance was significantly explained by the botanical origin (83.67% of variability). A high statistically significant effect was also found for the crystallization effect (15.45% of variance).

Table 11 shows the mean visual, texture, and overall liking scores (±SEM) per product, measured on a 9-point hedonic scale.

ANOVA testing yielded significant differences in visual, texture and overall liking between the products (all *p* < 0.0001). Post hoc comparisons (Tukey’s HSD test) on visual liking showed that the two liquid honey samples were significantly more liked (score 7.33 and 7.13 for rape and citrus, respectively) than the other honey samples. Crystallized honey samples had lower scores; in particular, the citrus sample showed the lowest significant score (3.38). For texture liking, the two liquid honey and the citrus creamy samples showed significantly higher scores (6.90, 6.88 and 6.51 for rape liquid, citrus liquid and citrus creamy samples, respectively) than the other honey samples. Crystallized samples had the significantly lowest scores (3.25 and 3.28 for citrus and rape sample). There were significant differences in the overall liking for the different types of honey samples at the 5% significance level. Citrus honey samples showed significantly higher scores compared to the rape ones. In particular, the rape crystallized honey sample had the lowest score (mean scores of 4.38), while the citrus liquid and creamy honey samples had the highest scores (mean scores of 6.44 and 6.25, respectively). For the three rape honeys, no significant differences were found between the liquid and creamy samples. Consumption frequency did not affect visual, texture and overall liking.

#### 3.3.2. CATA Counts

The citation frequencies of visual/texture CATA terms used to describe the six honeys are presented in Table 12.

Cochran’s Q test highlighted significant differences (*p* ≤ 0.05) among the term’s citation frequencies meaning the consumers perceived different visual/texture characteristics among the products.

**Figure 7:**
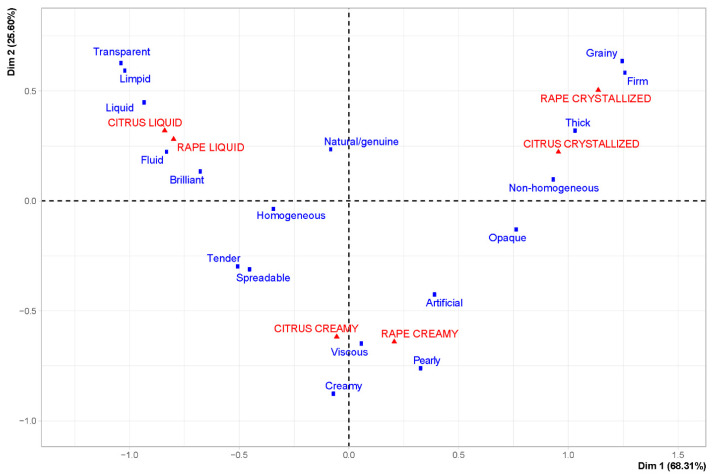
CA of visual/texture CATA terms and the six honeys evaluated.

CA also identified three main groups, characterized by different visual/texture characteristics (Figure 7), and corresponding to different crystallization levels.

The first one, composed of two liquid samples, described as transparent, limpid, liquid, fluid, brilliant and natural/genuine, was located at negative values of the first dimension and positive values of the second dimension. The crystallized samples were located at positive values of the first and second dimension and were described as firm, grainy, thick and non-homogeneous. Finally, the creamy samples were located at negative values of the second dimension and were described as viscous, pearly, and creamy, but also artificial, especially for rape honey. The terms tender and spreadable are associated with both creamy and liquid samples.

The citation frequencies of gustatory CATA terms used to describe citrus and rape honeys are reported in Table 13. 

Significant differences among samples were found in the citation frequency of twenty-three over twenty-six attributes: only three terms (persistent flavor, natural and pungent) were not significant. The pleasant flavor and off-flavor were mainly associated with the honey type: the first was significantly higher for citrus honey; the latter significantly described the rape honey. Terms related to the aromatic characterization, such as floral and fruity, were significantly related to both the monofloral type and the crystallization level. A low crystallization level was associated with higher floral citation frequencies in both citrus and rape samples, with the citrus samples showing higher frequencies compared to rape honeys. Higher crystallization levels showed lower trends of fruity perception, particularly in rape honeys characterized by higher fruity citation frequencies than rape samples. The crystallization level was correlated with a citation frequency decrease in the too sweet term. Liquid samples were characterized by higher sweetness perception. Terms related to the texture characteristics as creamy, firm, floury, liquid, tender and rough were significantly related to the rheological characteristics of the honey samples. Firm and rough were clearly associated to both the crystallized honey samples. The reduction of the crystallization level for both the honey types caused a decrease in the floury citation frequency and an increase in the liquid citation frequency. The term tender showed a strong association with the liquid and creamy samples of both honey types and a significant difference between the crystallized citrus and rape honey samples. The creamy citation frequency was significantly higher in both the creamy honey samples and lower in the crystallized honey samples, but no significant difference was found between liquid and crystallized citrus samples. The term big crystals was associated with the crystallized honey samples. The citrus crystallized honey sample had a significantly lower frequency compared to the rape crystallized sample, but significantly higher than the creamy and liquid. The fine crystals citation frequency was significantly higher in the creamy and crystallized citrus samples. The term melting, a meta-descriptor, was significantly associated with both citrus and rape creamy samples.

**Figure 8:**
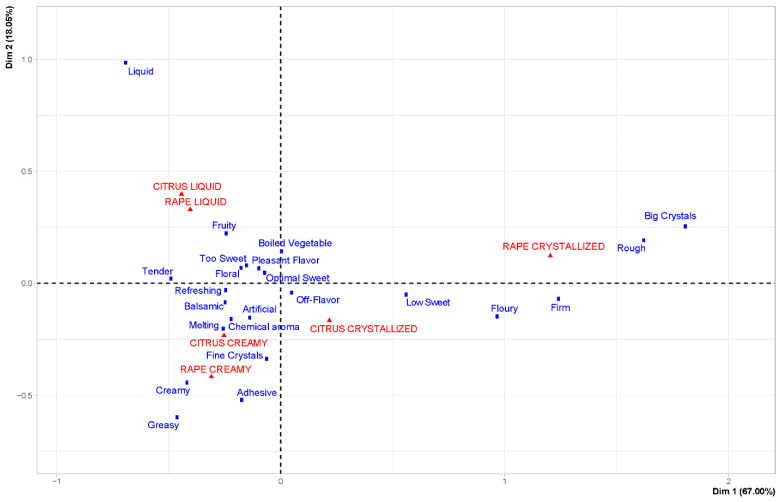
CA of gustatory CATA terms and the six honeys evaluated. Only statistically significant terms were used.

CA on the gustatory CATA citation frequencies is reported in Figure 8.

Liquid and creamy samples were sorted into groups according to their crystallization level. The citrus crystallized sample was separated from the rape crystallized ones being mainly described with big crystals and rough.

## 4. Discussion and Conclusions

In this study, the sensory analysis and consumer acceptance of honeys with different botanical origins and crystallization states have been investigated.

The rheological analysis of citrus and rape honey samples clearly showed different crystallization states (liquid, creamy and crystallized). The differences were not only related to the botanical origin, but also to different granulation levels. The intermediate consistency (creamy) for both monofloral honey types was generally caused by the presence of very small crystals. Enthalpy values do not give information about the dimensions and morphology of the crystals or about the uniformity of granulation in the sample. Nevertheless, according to Lupano [40], the shape of the melting peak and the melting temperature are related to the shape of the crystals, connected in turn to the conditions in which the granulation occurs. Tomaszewska-Gras et al. [42] observed in the range of 40–70 °C the presence of a broad and clear peak related to the solute–solute transition with remarkable differences in shape among honey types, but did not verify the reason behind this difference. Al-Habsi et al. [41] compared thermograms of four different types of honey, also observing the presence of complex peaks related to the melting of crystals. This complexity was attributed to the variability of the crystallized material.

The textural properties of the two creamy samples are very similar, although the microscopic images seemed different.

Sensory analysis performed by the expert panel to significantly discriminate the two monofloral honey types, demonstrating that the botanical origin determines honey flavor perception as reported in previous papers [43,44,45]. Citrus honeys were mainly characterized by floral flavor, differently from rape honeys described by vegetal, chemical and animal descriptors. The ANOVA factorial design showed the floral attribute had a moderately significant effect on the crystallization parameter (4.03% of variance). Moreover, the sweetness had a moderate effect on the crystallization state (24.44% of variance) and was mainly related to the botanical origin (72.97% of variance). Citrus samples were generally perceived as sweeter as confirmed by the higher fructose/glucose ratio in citrus honey. The impact of crystallization on the sweetness perceived was confirmed by a lower sweet intensity as the crystallization increased. The firmness perceived by the expert panel completely accounted for the crystallization effect (99% of variance), whereas the grainy attribute was equally explained by the botanical origin, crystallization and their relative interaction. The crystallization state affected the honey sensory perceptions; indeed, looking at the citrus samples, crystallization caused distinct taste and flavor dynamic perceptions. This paper is the first to report the influence of crystallization on taste and flavor dynamic evolutions. Indeed, liquid samples were significantly sweeter than crystallized ones. Moreover, firmness and grainy showed a relation with the crystallization state, with higher levels for creamy and crystallized samples, as expected. In terms of flavor perception, the floral attribute, characterizing the citrus honey, showed a lower value in the liquid sample. The TDS curve confirmed this perception; indeed, in the liquid samples, a significant peak of the warm attribute appeared in the last part of the floral dominance.

The DA did not show any statistically significant difference among the three crystallization states in the overall olfactory intensity. Whereas there was a clear difference in the dynamic perception; indeed, the TI highlighted a significantly lower AUC in the liquid sample. At the same time, in the crystallized sample, the time to reach the intensity peak was longer than in the other samples. These dynamics are in agreement with the lower floral intensity perception highlighted in the liquid sample by the DA. The citrus honeys showed a simple sensory characterization compared to the complex rape honey sensory description. The DA of the three rape samples showed no significant differences for any of the sensory characteristics evaluated except for firmness and grainy, clearly related to the different crystallization states.

The flavor dynamic evolution pointed out a correlation between the flavor perception and crystallization state. In the creamy sample, the TDS curve showed the animal dominance for the first 20 s, followed by vegetal reaching the significance level two times: at 25 s, and at 70–75 s. This long perception was confirmed by the higher level of the TI parameters (IMax, AUC and Dur), indicating a stronger and longer flavor stimulus. The rape liquid sample was characterized by animal flavor as well. Indeed, the TDS curve highlighted two distinct peaks, at the beginning of the evaluation (10 s), and at 50 s. In this period, two more flavors, vegetal and fruity, reached the significance level. In the crystallized rape sample, only animal and fruity reached the significance level with a dominance rate just above the limit. Moreover, chemical, vegetal and warm showed dominance rates higher than the chance level. This unclear flavor dynamic evolution trend of the crystallized sample made the sensory characterization by the expert panel difficult and can be related to the non-homogeneous crystallization, with crystals forming bigger structures together with isolated crystals compared to the creamy and liquid sample. Indeed, the perceived flavor, an unquestionable key component of food flavor, not only depends on the type and concentration of volatile compounds present in food, but is also influenced by the presence of certain food components (e.g., sweetener) and food textures [46,47]. As reported by Guinard and Mazzuchelli [15], Szczesniak [16], Tosi et al., [17], Saxena et al. [18], and Ajlouni and Sujirapinyokul [19], honey’s crystallization is an important factor influencing consumers’ preferences, making it less appealing to consumers, who prefer liquid and/or transparent honey. Our data, related to visual, texture and overall liking of honeys at different crystallization state, confirmed these results. Both citrus and rape samples showed consumers’ preference for liquid samples, with liking decreasing as the crystallization state increased. The CATA data related to visual/texture properties were able to identify the attributes that better describe the samples. The terms proposed were correctly used and understood by consumers. The gustatory CATA data reported an increase of some positive terms (fruity, floral, and pleasant) in liquid and creamy samples. The crystallization caused a decrease in the too sweet citation frequency. Moreover, a sweetness-increased trend was observed in liquid samples as confirmed by the expert panel.

Finally, our work studied two different monofloral honeys in three different crystallization states, showing that the botanical origin, together with the crystallization state, are the main factors affecting honey sensory features. The crystallization state plays an important role defining, not only texture characteristics, but also honey flavors and taste perception. Indeed, honeys characterized by a simple flavor profile (e.g., citrus) have higher flavor and taste perceptions compared to complex flavor profile honeys (e. g. rape). The sensory profiles defined by the expert panel, in line with the consumers’ liking and perceptions, were shown to be affected by the crystallization state, highlighting its essential discriminative power to classify honey.

The main novelty of this research was to analyze the sensory properties of the same honey related to three different crystallization states and compare them with consumers’ acceptance. Honey producers should take into account that controlled crystallization is a valuable instrument to influence consumers’ acceptance, remembering that botanical origin also plays an important role in honey sensory appreciation. Indeed, consumers widely appreciated citrus honey characterized by floral, fruity, sweet notes and a more homogeneous crystallization, but producers and distributors can promote rape honey, highlighting its peculiar texture and flavor complexity. They could rely on the stronger and longer flavor perception turning the rape honey’s non–homogenous crystallization into a marketing strategy leading to honey success. Furthermore, they can modulate honey crystallization, obtaining the most preferred crystallization state by the consumers.

In this research, a possible limitation lies in the restricted number of monofloral honeys involved; therefore, the two honeys chosen for the study represent two different acceptability levels related to different aromatic properties. 

This study opens up to further research that will help cover a wider range of honey samples with different botanical origins and sensory profiles, gaining more information on the crystallization state, sensory properties and consumer’s acceptance correlation. 

## Figures and Tables

**Table 1:** Honeys’ analysis. Determination of saccharides content (fructose, glucose, sucrose, turanose, and maltose) through HPLC method (DIN 10758:1997-05) and water content. Expanded uncertainty is expressed with a coverage factor of two and a confidence limit of 95%.

Parameters	Citrus Honey	Rape Honey
Fructose% (*w*/*w*)	41.0 ± 4.5	35.5 ± 3.9
Glucose% (*w*/*w*)	33.1 ± 4.3	37.6 ± 4.8
Sucrose% (*w*/*w*)	0.9 ± 0.1	<0.5
Turanose% (*w*/*w*)	1.5 ± 0.2	0.9 ± 0.1
Maltose% (*w*/*w*)	2.6 ± 0.3	0.7 ± 0.1
Fructose/Glucose ratio% (*w*/*w*)	1.24	0.94
Invert Sugar (Fructose + Glucose)% (*w*/*w*)	74.1 ± 8.8	73.1 ± 8.7
Water content%	17.1	18.7

**Table 2:** Melting enthalpy (ΔH, J/g) and temperature (Tm, °C) as obtained by differential scanning calorimetry (±SEM). Values with different letters correspond to statistical differences (Tukey’s HSD test).

Product	Citrus Honey		Rape Honey	
	ΔH	Tm	ΔH	Tm
Liquid	-	-	-	-
Creamy	21.81 b ± 0.16	53.0 a ± 0.5	31.5 a ± 1.9	65.21 a ± 0.56
Crystallized	22.70 b ± 1.10	52.3 a ± 0.5	31.4 a ± 1.2	57.51 b ± 0.10

**Table 3:** Water activity (*a_w_*) and textural parameters of honey samples (±SEM): hardness (N), adhesivity (N s), and color indexes (brightness (L*), red index (a*) and hue angle (h°)).

Product	*a_w_*	Hardness (N)	Adhesivity (N s)	L*	a*	h°
	Citrus honey					
Liquid	0.515 ± 0.004	0.039 ± 0.013	−0.200 ± 0.084	75.31 ± 0.04	−1.51 ± 0.06	92.55 ± 0.12
Creamy	0.550 ± 0.003	0.023 ± 0.001	−0.035 ± 0.001	68.91 ± 0.47	0.14 ± 0.36	89.68 ± 0.79
Crystallized	0.546 ± 0.004	10.287 ± 1.753	−9115 ± 1.193	75.31 ± 0.73	0.73 ± 0.07	88.27 ± 0.21
	Rape honey					
Liquid	0.530 ± 0.002	0.031 ± 0.006	−0.128 ± 0.045	70.97 ± 0.11	1.48 ± 0.18	88.16 ± 0.19
Creamy	0.576 ± 0.006	0.026 ± 0.001	−0.130 ± 0.009	76.01 ± 0.16	2.78 ± 0.05	83.41 ± 0.12
Crystallized	0.586 ± 0.001	5.319 ± 8.821	−2500 ± 4.315	70.84 ± 0.04	4.15 ± 0.63	82.60 ± 1.22

**Table 4:** Sensory characterization by the expert panel.

	Citrus Honey			Rape Honey		
Attribute	Liquid	Creamy	Crystallized	Liquid	Creamy	Crystallized
Global olfactory intensity **	4.63 ± 0.35 b	4.97 ± 0.21 b	4.71 ± 0.28 b	6.09 ± 0.39 a	5.75 ± 0.30 ab	5.73 ± 0.22 ab
Floral ***	2.18 ± 0.56 b	3.33 ± 0.37 a	3.22 ± 0.46 a	0.18 ± 0.10 c	0.66 ± 0.47 c	0.46 ± 0.34 c
Fruity ns	1.58 ± 0.27	1.96 ± 0.44	1.47 ± 0.28	1.88 ± 0.56	1.59 ± 0.43	2.03 ± 0.53
Warm ns	1.54 ± 0.39	1.48 ± 0.46	1.95 ± 0.32	1.84 ± 0.41	1.88 ± 0.43	1.22 ± 0.30
Aromatic ns	0.43 ± 0.21	0.94 ± 0.41	0.55 ± 0.24	0.73 ± 0.33	0.66 ± 0.27	0.66 ± 0.24
Chemical *	0.06 ± 0.04 b	0.15 ± 0.08 ab	0.15 ± 0.08 ab	0.85 ± 0.44 a	0.28 ± 0.19 ab	0.79 ± 0.25 a
Vegetal ***	0.65 ± 0.20 b	0.75 ± 0.26 b	0.81 ± 0.32 b	3.25 ± 0.55 a	3.24 ± 0.36 a	2.80 ± 0.48 a
Animal ***	0.32 ± 0.31 b	0.01 ± 0.00 b	0.09 ± 0.06 b	3.08 ± 0.83 a	2.72 ± 0.75 a	2.36 ± 0.66 a
Sweetness ***	7.00 ± 0.33 a	6.28 ± 0.57 ab	5.27 ± 0.58 bc	5.48 ± 0.57 bc	5.21 ± 0.50 bc	4.73 ± 0.43 c
Acidity ns	2.21 ± 0.37	2.73 ± 0.53	2.49 ± 0.56	2.87 ± 0.66	2.98 ± 0.59	3.01 ± 0.74
Bitterness ns	0.19 ± 0.19	0.29 ± 0.20	0.33 ± 0.23	0.11 ± 0.07	0.11 ± 0.08	0.26 ± 0.21
Saltiness ns	0.03 ± 0.03	0.05 ± 0.05	0.06 ± 0.06	0.22 ± 0.12	0.13 ± 0.06	0.09 ± 0.05
Firmness ***	1.23 ± 0.52 b	1.23 ± 0.36 b	8.07 ± 0.42 a	0.86 ± 0.34 b	1.83 ± 0.57 b	8.83 ± 0.23 a
Grainy ***	0.00 ± 0.00 c	0.19 ± 0.08 bc	0.42 ± 0.15 b	0.00 ± 0.00 c	0.15 ± 0.08 bc	8.13 ± 0.39 a

Mean intensity of each attribute of the monofloral honey samples (±SEM). Means with different letters correspond to statistical differences (Tukey’s HSD test). Asterisks correspond to different *p*-value levels: * 0.05, ** 0.01 or *** 0.001; ns = not significant.

**Table 5:** ANOVA factorial design. Variability is expressed as % of the total sum of the squares for sensory attributes.

Attribute	Botanical Origin	Crystallization	Botanical Origin X Crystallization
Global olfactory intensity	96.04 **	3.93 ns	0.03 ns
Floral	94.84 ***	4.03 (.)	1.13 ns
Fruity	21.97 ns	1.44 ns	76.59 ns
Warm	1.98 ns	13.59 ns	84.43 ns
Aromatic	6.34 ns	32.44 ns	61.22 ns
Chemical	79.71 **	8.24 ns	12.05 ns
Vegetal	99.18 ***	0.03 ns	0.79 ns
Animal	98.46 ***	1.19 ns	0.35 ns
Sweetness	72.97 **	24.44 (.)	2.59 ns
Acidity	83.02 ns	11.45 ns	5.53 ns
Bitterness	59.37 ns	32.25 ns	8.38 ns
Saltiness	78.54 ns	5.33 ns	16.13 ns
Firmness	0.46 ns	99.00 ***	0.54 ns
Grainy	31.29 ***	37.11 ***	31.60 ***

Significant *F*-values levels are indicated as follows: (.) 0.10, ** 0.01, *** 0.001; ns = not significant.

**Table 6:** Time intensity parameters (±SEM) for the citrus honey overall olfactory intensity.

Product	IMax	Tmax	AUC	Dur
Liquid	59.0 ± 3.25 ns	8.0 ± 0.55 b	1577 ± 162 b	49.7 ± 7.18 ns
Creamy	64.0 ± 3.58	9.4 ± 0.58 b	2396 ± 694 a	68.2 ± 15.8
Crystallized	63.2 ± 4.23	12.0 ± 1.75 a	2623 ± 545 a	77.3 ± 13.2

(Imax: the highest intensity on TI record; Tmax: time to reach peak intensity; AUC: total area under the time-intensity curve; Dur: time from onset to return to baseline). Different letters represent significant differences among treatments according to Tukey’s HSD test (*p* < 0.05).

**Table 7:** Time intensity parameters (±SEM) for the rape honey overall olfactory intensity.

Product	IMax	Tmax	AUC	Dur
Liquid	59.6 ± 3.16 b	7.4 ± 0.95 b	1860 ± 243 b	58.8 ± 10.2 b
Creamy	67.4 ± 2.33 a	9.6 ± 0.94 a	2552 ± 284 a	73.0 ± 5.41 a
Crystallized	59.0 ± 3.93 b	9.8 ± 0.75 a	1794 ± 192 b	55.2 ± 13.9 b

(Imax: the highest intensity on TI record; Tmax: time to reach peak intensity; AUC: total area under the time-intensity curve; Dur: time from onset to return to baseline). Different letters represent significant differences among treatments according to Tukey’s HSD test (*p* < 0.05).

**Table 8:** Preferred honey consumption mode scored on a 9-point scale (1 = disagree completely; 9 = agree completely).

How Do I Consume Honey?	Agreement Level
As is	3.83 ± 0.20 bc
Spreaded on a slice of bread	4.06 ± 0.23 b
As a sweetener	5.64 ± 0.24 a
As an ingredient in food	3.16 ± 0.20 c
In food pairing	4.44 ± 0.21 b

Means (±SEM) followed by different letters correspond to statistical differences (Tukey’s HSD test).

**Table 9:** Motivational consumption scored on a 9-point scale (1 = disagree completely; 9 = agree completely).

Why Do I Consume Honey?	Agreement Level
Because I like it	7.44 ± 0.15 a
For therapeutic use	4.98 ± 0.22 b
Because is a “natural/genuine” product	5.54 ± 0.21 b

Means (±SEM) followed by different letters correspond to statistical differences (Tukey’s HSD test).

**Table 10:** ANOVA factorial design.

Liking	Botanical Origin	Crystallization	Botanical Origin X Crystallization
Visual liking	0.09 ns	90.54 ***	9.37 *
Texture liking	7.00 *	85.54 ***	7.46 *
Overall liking	83.67 ***	15.45 ***	0.87 ns

Variability is expressed as % of the total sum of the squares for visual liking, texture liking and overall liking. Significant *F*-values levels are indicated as follows: * 0.05, and *** 0.001; ns = not significant.

**Table 11:** Mean liking scores on a 9-point hedonic scale (±SEM).

Product	Visual Liking	Texture Liking	Overall Liking
Citrus Liquid	7.13 ± 0.12 a	6.88 ± 0.13 a	6.44 ± 0.15 a
Citrus Creamy	5.68 ± 0.17 b	6.51 ± 0.15 a	6.25 ± 0.16 a
Citrus Crystallized	3.38 ± 0.17 d	3.25 ± 0.17 c	5.80 ± 0.18 ab
Rape Liquid	7.33 ± 0.12 a	6.90 ± 0.14 a	5.47 ± 0.18 b
Rape Creamy	4.22 ± 0.19 c	4.65 ± 0.19 b	5.11 ± 0.19 b
Rape Crystallized	4.00 ± 0.18 cd	3.28 ± 0.17 c	4.38 ± 0.19 c

Means followed by different letters correspond to statistical differences (Tukey’s HSD test).

**Table 12:** Cochran’s Q tests for the citation frequencies of the visual/texture CATA terms.

Term	Citrus Liquid	Citrus Creamy	Citrus Cristallized	Rape Liquid	Rape Creamy	Rape Cristallized	Total Terms
Creamy ***	22 b	99 a	10 c	25 b	89 a	3 d	248
Pearly ***	9 c	88 a	35 b	4 c	83 a	22 b	241
Homogeneous ***	95 a	83 a	35 b	95 a	46 b	19 c	373
Brilliant ***	65 a	30 b	5 c	72 a	21 b	4 c	197
Viscous ***	21 b	63 a	17 b	21 b	74 a	14 b	210
Spreadable ***	81 a	95 a	10 c	88 a	70 b	2 d	346
Tender ***	50 ab	66 a	3 c	64 a	39 b	1 c	223
Natural/genuine **	40 a	26 bc	20 cd	39 ab	14 d	30 abc	169
Thick ***	3 c	28 b	113 a	7 c	37 b	114 a	302
Fluid ***	106 a	39 b	1 d	110 a	20 c	1 d	277
Transparent ***	105 a	2 b	1 b	96 a	1 b	2 b	207
Opaque ***	3 c	72 b	99 a	8 c	74 b	91 a	347
Artificial **	7 c	16 bc	21 b	9 c	40 a	14 bc	107
Limpid ***	99 a	4 b	2 b	94 a	2 b	1 b	202
Liquid ***	80 a	13 b	1 c	83 a	6 b	2 c	185
Firm ***	0 c	9 b	121 a	1 c	16 b	120 a	267
Grainy ***	0 d	10 c	22 b	1 d	12 bc	104 a	149
Non-homogeneous ***	2 c	5 c	38 b	3 c	43 ab	54 a	145
Total Terms	788	748	554	820	687	598	4195

Significant *p*-value levels are indicated as follows: ** *p* ≤ 0.01, *** *p* ≤ 0.001, and “ns” for no significant differences (*p* > 0.05). Different letters within each row correspond to statistical differences according to the sign test (*p* ≤ 0.05).

**Table 13:** Cochran’s Q tests for the citation frequencies of the gustatory CATA terms.

Term	Citrus Liquid	Citrus Creamy	Citrus Crystallized	Rape Liquid	Rape Creamy	Rape Crystallized	Total Terms
Adhesive ***	12 c	23 b	24 b	10 c	47 a	10 c	126
Pleasant Flavor ***	75 a	70 a	71 a	51 b	40 b	41 b	348
Persistent Flavor ns	33	39	33	39	29	22	195
Off-Flavor ***	5 b	9 b	8 b	29 a	29 a	22 a	102
Balsamic *	22 a	23 a	28 a	20 a	22 a	6 b	121
Chemical flavor *	6 b	11 ab	9 ab	16 a	18 a	6 b	66
Creamy ***	32 b	83 a	24 b	25 b	72 a	4 c	240
Fine Crystals ***	10 b	35 a	27 a	9 b	15 b	10 b	106
Big Crystals ***	0 c	2 c	16 b	0 c	1 c	107 a	126
Optimal Sweet **	77 a	63 ab	69 ab	53 b	55 b	49 b	366
Firm ***	0 b	2 b	64 a	0 b	3 b	76 a	145
Floury ***	1 d	28 b	47 a	3 d	11 c	81 a	171
Floral ***	51 a	44 ab	46 ab	32 bc	26 cd	19 d	218
Melting ***	38 b	56 a	45 ab	38 b	58 a	15 c	250
Fruity ***	50 a	23 b	30 b	29 b	23 b	14 b	169
Liquid ***	95 a	14 b	1 c	91 a	7 b	1 c	209
Artificial *	13 b	19 ab	21 ab	20 ab	32 a	13 b	118
Tender ***	70 a	71 a	23 b	63 a	65 a	5 c	297
Natural/Genuine ns	46	40	34	34	24	34	212
Low Sweet **	5 c	8 bc	14 b	11 bc	16 b	32 a	86
Pungent ns	11	11	11	14	14	5	66
Refreshing *	30 a	25 a	19 ab	18 ab	24 a	11 b	127
Rough ***	0 d	7 c	18 b	2 cd	2 cd	99 a	128
Too Sweet *	42 ab	43 ab	32 b	54 a	41 ab	30 b	242
Greasy ***	6 b	17 b	12 b	19 b	52 a	0 c	106
Boiled Vegetable ***	1 d	1 d	4 cd	22 a	14 ab	11 bc	53
Total Terms	731	767	730	702	740	723	4393

Significant *p*-value levels are indicated as follows: * *p* ≤ 0.05, ** *p* ≤ 0.01, *** *p* ≤ 0.001, and “ns” for no significant differences (*p* > 0.05). Different letters within each row correspond to statistical differences according to the sign test (*p* ≤ 0.05).

## Data Availability

The datasets generated for this study are available on request to the corresponding author.
